# Mitochondrial DNA suggests at least 11 origins of parasitism in angiosperms and reveals genomic chimerism in parasitic plants

**DOI:** 10.1186/1471-2148-7-248

**Published:** 2007-12-21

**Authors:** Todd J Barkman, Joel R McNeal, Seok-Hong Lim, Gwen Coat, Henrietta B Croom, Nelson D Young, Claude W dePamphilis

**Affiliations:** 1 Department of Biological Sciences, Western Michigan University, Kalamazoo, MI, 49008, USA; 2 Department of Biology and Institute of Molecular Evolutionary Genetics, Pennsylvania State University, University Park, PA 16802, USA; 3 Department of Plant Biology, University of Georgia, Athens, GA 30602, USA; 4 Department of Biology, University of the South, Sewanee, TN 37383, USA; 5 Department of Microbiology, 442F Morrill IVN, U Mass Amherst, Amherst, MA 01536, USA

## Abstract

**Background:**

Some of the most difficult phylogenetic questions in evolutionary biology involve identification of the free-living relatives of parasitic organisms, particularly those of parasitic flowering plants. Consequently, the number of origins of parasitism and the phylogenetic distribution of the heterotrophic lifestyle among angiosperm lineages is unclear.

**Results:**

Here we report the results of a phylogenetic analysis of 102 species of seed plants designed to infer the position of all haustorial parasitic angiosperm lineages using three mitochondrial genes: *atp1, coxI*, and *matR*. Overall, the mtDNA phylogeny agrees with independent studies in terms of non-parasitic plant relationships and reveals at least 11 independent origins of parasitism in angiosperms, eight of which consist entirely of holoparasitic species that lack photosynthetic ability. From these results, it can be inferred that modern-day parasites have disproportionately evolved in certain lineages and that the endoparasitic habit has arisen by convergence in four clades. In addition, reduced taxon, single gene analyses revealed multiple horizontal transfers of *atp1 *from host to parasite lineage, suggesting that parasites may be important vectors of horizontal gene transfer in angiosperms. Furthermore, in *Pilostyles *we show evidence for a recent host-to-parasite *atp1 *transfer based on a chimeric gene sequence that indicates multiple historical xenologous gene acquisitions have occurred in this endoparasite. Finally, the phylogenetic relationships inferred for parasites indicate that the origins of parasitism in angiosperms are strongly correlated with horizontal acquisitions of the invasive *coxI *group I intron.

**Conclusion:**

Collectively, these results indicate that the parasitic lifestyle has arisen repeatedly in angiosperm evolutionary history and results in increasing parasite genomic chimerism over time.

## Background

The parasitic lifestyle has evolved repeatedly in nearly every major lineage of life, and in the broad sense includes brood parasitism, social parasitism, genomic parasitism, and nutritional parasitism [1,2]. Among plants, nutritional parasites obtain water and nutrients directly from their photosynthetic host plant through a specialized feeding structure, the haustorium, which is attached to either host shoots or roots [3]. These plants include both hemiparasites (parasites with the ability to photosynthesize) and holoparasites (those that cannot photosynthesize) [3]. While both hemi- and some holoparasites grow largely exterior to the host, certain holoparasites grow nearly completely embedded within the host plant tissues as endoparasites, emerging only during sexual reproduction [3,4]. Though most parasites can be classified according to their photosynthetic status and the nature of their interactions with their hosts, insight into the evolution of parasitic traits has been hampered by the lack of a broad phylogenetic perspective.

The parasitic lifestyle is thought to have evolved 8 [3] to 11 [5] times in flowering plants, but comprehensive phylogenetic analyses have never been performed to investigate the evolutionary frequency and pattern of the shift to heterotrophy in angiosperms. The lack of a robust phylogenetic hypothesis for parasitic angiosperms has also hampered studies of genome evolution [6] and the inference of ancestral conditions that may have promoted the evolution of the haustorial parasitic lifestyle. Furthermore, although considerable progress has been made towards the molecular systematics of many parasitic plants [5,7-12], several parasites have obscure positions within angiosperm phylogeny, accounting for 7 of 18 unplaced taxa in the recent molecularly-based ordinal classification of flowering plants [13].

Classifying parasitic plants using morphological characters has long been difficult because of the extreme reduction or alteration of vegetative and floral morphology that occurs in holoparasitic lineages [3,14]. The primary challenge associated with inferring the phylogenetic placement of many parasites using molecular data in a global angiosperm context is spurious long-branch attraction [15] caused by their highly divergent DNA sequences [5,11]. Furthermore, the apparent loss of photosynthetic and other genes that have been commonly used to study flowering plant phylogeny [5,6,16] has prevented the inclusion of many parasites in otherwise comprehensive studies [13,17,18]. A possible solution to these phylogenetic problems is through the study of plant mtDNA, which is retained regardless of photosynthetic ability and has proven useful for determining the phylogenetic affinities of some parasitic plants [10-12]. To infer the number of origins and distribution of parasitism in a global angiosperm phylogenetic context we used three mtDNA genes:*atp1*, *coxI*, and *matR*.

While mtDNA may offer several advantages for the study of parasitic plants, its use necessitates careful consideration of the possibility of horizontal gene transfer (HGT) [19-26]. Of particular relevance to our study are reports that mitochondrial genes may be horizontally transferred between hosts and parasites [21,23,25,27]. Also, the invasive mitochondrial *coxI *group I intron (which is lacking from most plants) has been independently, horizontally acquired from unknown vectors in various flowering plants [28]. One consequence of such transfers is that parasites may appear closely related to their hosts, thereby obscuring their true phylogenies; however, if such occurrences are relatively uncommon, the majority of loci should correctly predict the phylogenetic positions of most parasites. Our multigene approach coupled with broad angiosperm ordinal sampling will allow us to estimate the number of parasitic plant origins in flowering plant phylogeny, interpret potential horizontal transfers of foreign mtDNA into parasitic plant genomes and discern whether parasites are more likely to horizontally acquire DNA than non-parasites.

## Results

### Phylogenetic placement of parasites

**Figure 1:**
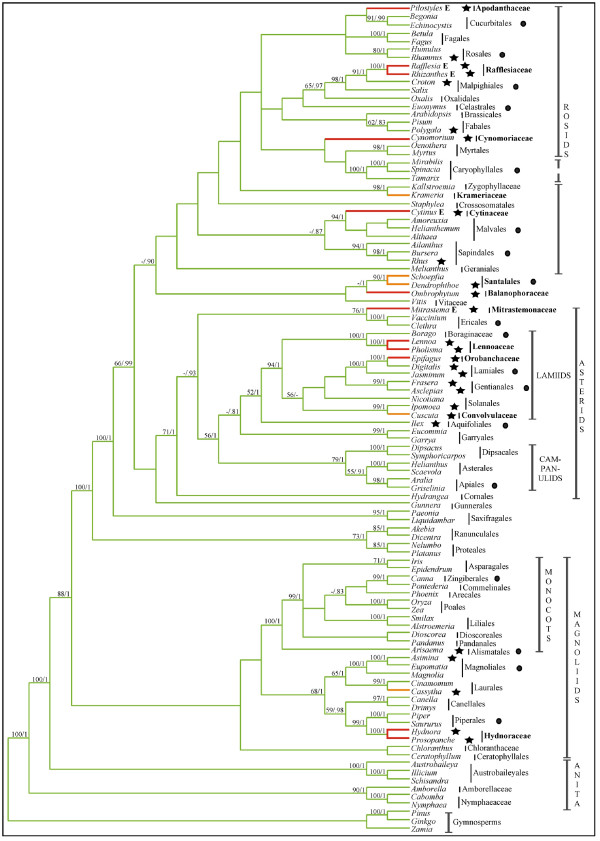
**Phylogenetic tree estimated from 3 combined mt genes indicates at least 11 origins of parasitism in angiosperm phylogeny**. Single best tree estimated from ML analysis of combined *atp1 *+ *coxI *+ *matR *mtDNA sequence data (-lnL = 43115.38). ML bootstrap support/Bayesian posterior probability values (BP/PP) are shown above all nodes with values >50/0.8. Green branches are non-parasitic, orange branches are mostly hemiparasitic, and red branches are holoparasitic lineages. A star next to a taxon represents the presence of the *coxI *intron in the sampled species. A filled circle next to an order or family represents the presence of the mitochondrial *coxI *intron based on literature reports [28].

**Figure 2:**
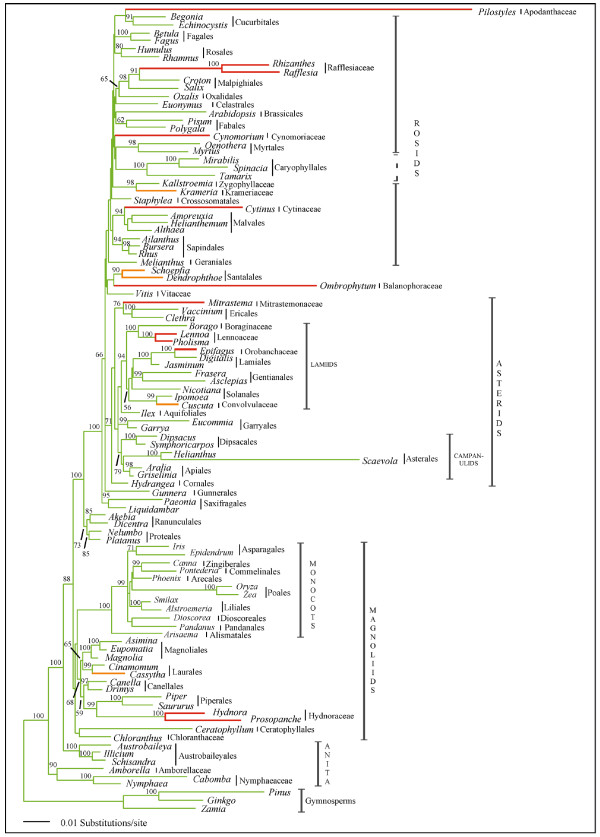
**Estimated branch lengths for combined 3 mt gene phylogenetic analysis**. Single best tree from Fig. 1 with branch lengths estimated from ML analysis of combined *atp1 *+ *coxI *+ *matR *mtDNA sequence data (-lnL = 43115.38). Green branches are non-parasitic, orange branches are mostly hemiparasitic, and red branches are holoparasitic lineages. Some parasites have long estimated branch lengths for these 3 combined genes; however, even some non-parasites, like *Scaevola*, have long branch lengths as well. ML bootstrap support values (BP) are shown above all nodes with values >50.

Analyses of combined *atp1*, *coxI*, and *matR *sequence data (4,019 aligned base pairs; TreeBASE accession S1932) were undertaken because initial single gene analyses showed that there were no strongly supported conflicting nodes between trees estimated from separate datasets (trees not shown). Maximum likelihood analyses of the 3 gene combined dataset resulted in a single best tree (-lnL = 43115.38) that represents the first molecular phylogenetic placement of all parasitic lineages within a global angiosperm context (Fig. 1 &2). This mtDNA phylogenetic tree shows largely congruent relationships among non-parasitic angiosperms compared to independent analyses of chloroplast and nuclear sequence data [13,17,18]. Highly supported placements were obtained for nine parasite lineages that corroborate earlier focussed studies: Hydnoraceae with Piperales (BP = 99; PP = 1.0) [10], *Cassytha *with Laurales (BP = 99; PP = 1.0) [8], *Cuscuta *with Solanales (BP = 99; PP = 1.0) [9], Orobanchaceae with Lamiales (BP = 100; PP = 1.0) [7], Lennoaceae with Boraginaceae (BP = 100; PP = 1.0) [29], Mitrastemonaceae with Ericales (BP = 76; PP = 1.0) [11], Cytinaceae with Malvales (BP = 94; PP = 1.0) [23], Krameriaceae with Zygophyllaceae (BP = 98; PP = 1.0) [30], and Rafflesiaceae with Malpighiales (BP = 98; PP = 1.0) [11,31] (Fig. 1). Lesser support was obtained for Balanophoraceae with Santalales (BP < 50; PP = 1.0) (a relationship suggested in previous analyses [12]) and Apodanthaceae with Cucurbitales (BP < 50; PP = 0.66), although an Apodanthaceae + Cucurbitales relationship is further supported by a unique shared 3 bp (one codon) insertion at bp 341 in *matR *(coordinates from *Arabidopsis thaliana *NC001284). Combined analyses were entirely unclear about the placement of *Cynomorium*; however, sequence data of *matR*-alone suggest this parasite is related to Saxifragales (BP = 72; PP = 1.0; tree not shown), in agreement with previous analyses [12,32]. In contrast, combined *atp1 *and *coxI *analyses support a placement of *Cynomorium *with Sapindales (BP < 50; PP = 1.0; tree not shown).

Within this phylogenetic framework, it appears that haustorial parasitism has arisen at least 12 independent times as indicated by the orange (mostly hemiparasitic) and red (holoparasitic) branches (Fig. 1). It is not clear whether parasitism arose once in the ancestor of Balanophoraceae + Santalales or if the parasitic lifestyle independently evolved in the two lineages because the earliest diverging branches of Santalales are not parasitic and it is unlikely that parasitism is a reversible trait [33]. Thus, it is possible that there are actually 13 origins of parasitism implied by this tree (Fig. 1). Because some parasite phylogenetic positions did not receive high bootstrap support, we attempted to discern whether a hypothesis of fewer than 12 origins of parasitism could be rejected. Trees that constrained Apodanthaceae + Cynomoriaceae + Santalales + Balanophoraceae to be monophyletic in any of the three positions shown in Fig. 1 were rejected by the S-H test as significantly worse than the unconstrained tree of Fig. 1 (*P *< 0.05 in all cases). Trees that constrained the position of Apodanthaceae as sister to Cynomoriaceae or Santalales were significantly worse than the optimal tree of Fig. 1 (*P *< 0.05). Trees constraining the position of Santalales + Balanophoraceae with Apodanthaceae were significantly worse than the optimal tree (*P *< 0.05) while a position sister to Cynomoriaceae could not be rejected. Finally, trees that constrained the position of Cynomoriaceae-only to be sister to either Apodanthaceae or Santalales + Balanophoraceae were not significantly different from the optimal tree shown in Fig. 1. Thus, these data suggest that there were as few as 11, or as many as 13, origins of parasitism in angiosperm evolutionary history.

The phylogenetic tree shown in Fig. 1 also reveals a surprising feature of parasite evolution: endoparasitism has arisen in four independent lineages. These four lineages, marked by an "E" in Figure 1, include Apodanthaceae, Rafflesiaceae, Cytinaceae, and Mitrastemonaceae (hereafter referred to as "endoparasites"). Because these four endoparasite families have traditionally been included in Rafflesiaceae it was previously assumed that endoparasitism was uniquely derived [3], although several recent studies have shown that these families are not closely related [11,21,23,31]. Figure 1 clearly indicates that the endoparasites are not monophyletic. In fact, trees that constrained the endoparasite clade to be monophyletic in any of the four positions shown in Fig. 1 were rejected by the S-H test as significantly worse than the unconstrained tree of Fig. 1 (*P *< 0.05 in all cases).

### Putative horizontal transfer of *atp1*

**Figure 3:**
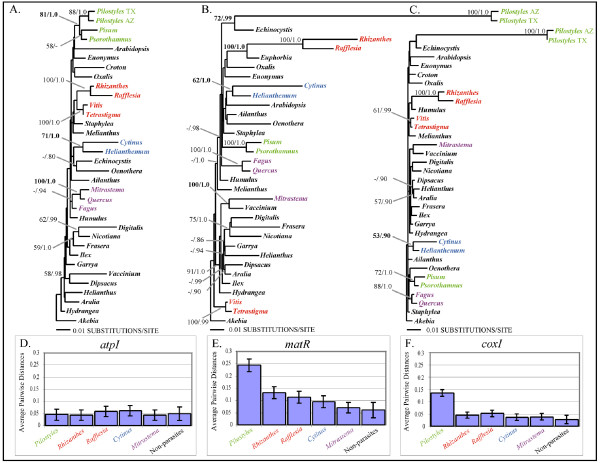
**Single gene analyses reveal potential cases of horizontal gene transfer of *atp1 *from host to parasite**. Comparison of phylogenetic relationships, gene-specific branch lengths (drawn proportionally) and corrected pairwise divergences (*K*) for representatives of all rosid and asterid orders for the three mt genes, *atp1, matR*, and *coxI*. Endoparasites and their host lineages are shown in matching colors. BP/PP values are shown above all nodes with values >50/0.8. A. Single most likely tree from *atp1*-only analysis (-lnL = 5136.56). B. Single most likely tree from *matR*-only analysis (-lnL = 8443.48). C. Single most likely tree from *coxI*-only analysis (-lnL = 4426.50). D-F. Average pairwise divergences for the endoparasite taxa relative to all non-parasites in the *atp1, matR*, and *coxI *datasets, respectively. Calculations of pairwise divergences shown in D-F, were made by comparing each single endoparasite to all non-parasites and all non-parasites to each other.

Because of unexpected phylogenetic relationships noted in preliminary analyses of *atp1 *(tree not shown), single gene studies using one representative for each rosid and asterid order, together with the four endoparasites, were performed. Figure 3 shows that analyses of *atp1*-alone suggest all four endoparasite lineages are closely related to their primary host lineages: *Pilostyles *(TX + AZ) with *Pisum *+ *Psorothamnus *(BP = 81; PP = 1.0), *Rafflesia *+ *Rhizanthes *with *Vitis *+ *Tetrastigma *(BP < 50; PP = 0.72), *Cytinus *with *Helianthemum *(BP = 71; PP = 1.0), and *Mitrastema *+ *Quercus *(BP = 100; PP = 1.0) with *Fagus *(BP < 50; PP = 0.94) (Fig. 3A). Although node-specific support for these parasite + host plant sister relationships is not high in most cases, it is extremely unlikely that they would all independently be related to their hosts by chance alone. In all of these parasites, *atp1 *has an intact open-reading frame and we have never found any evidence for additional copies of the gene using PCR. These results could indicate that all of these endoparasites are closely related to their primary host lineages; however, the *matR *and *coxI *single gene analyses (Fig. 3B & C), the combined three gene analyses (Fig. 1 &2), as well as recent studies [11,21,23,31] suggest otherwise for *Pilostyles*, *Rafflesia *+ *Rhizanthes*, and *Mitrastema*. In contrast, *Cytinus *does indeed appear to be related to Malvales with all three genes analyzed here (Fig. 3A–C). Host plant contamination cannot explain the results for *atp1 *because, in all cases, replication of our methods produced identical results and comparisons of sequences from the parasites to the host individuals from which they were collected, revealed numerous differences (Fig. 3A–C). Rather, these results suggest a different history for *atp1 *as compared to *coxI *and *matR *in *Pilostyles*, *Rafflesia *+ *Rhizanthes*, and *Mitrastema*, most likely caused by historical horizontal gene transfer (HGT) from their hosts. Conflicting histories for *atp1 *relative to *matR *+ *coxI *in these parasites are also inferred from statistically significant S-H test results that compared the unconstrained tree in Fig 3A to one which constrained the positions of the endoparasites to those of Fig. 1 (*P *< 0.05). However, when each parasite was analyzed singly, only the position of *Mitrastema *was statistically significant (*P *< 0.05). Furthermore, corrected pairwise divergences (*K*) and estimated branch lengths indicate substantial disparities in the level of divergence of *atp1 *compared to *coxI *and *matR *in these parasites relative to non-parasites (Fig. 3A–F). Specifically, levels of sequence divergence and branch lengths for *coxI *and *matR *in *Pilostyles *are ca. 4–5 times higher than in non-parasitic plants. However, in stark contrast, the level of sequence divergence for *atp1 *is indistinguishable between *Pilostyles *and non-parasites. This disparate pattern is also detectable to a lesser extent in *Rafflesia*, *Rhizanthes*, and *Mitrastema*. This observation is consistent with the expectation that recently transferred xenologous *atp1 *genes should be less divergent than native sequences that have resided for longer periods of time in the rapidly evolving parasitic plant genomes.

**Figure 4:**
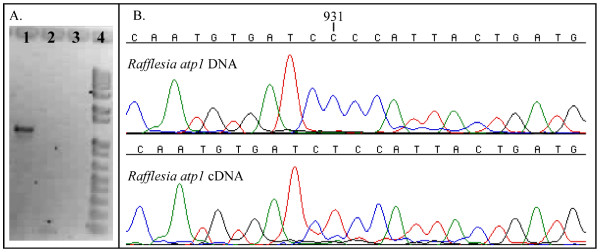
**Comparison of *atp1 *cDNA and DNA sequences in *Rafflesia cantleyi***. A. Agarose gel showing 1: RT-PCR results obtained using an initial reverse transcription step during thermalcycling, 2: RT-PCR results obtained without an initial reverse transcription step during thermalcycling, 3: RT-PCR results obtained without adding RNA to reaction but using an initial reverse transcription step during thermalcycling, and 4: 1 kb ladder. Results for lane 1 as compared to lane 2 indicate that cDNA was amplified from *Rafflesia *RNA. B. Comparisons of *Rafflesia atp1 *DNA and cDNA sequences. Position 931 appears to be RNA edited because a T was determined to be encoded in the cDNA while a C is encoded in the DNA.

To investigate whether the putatively horizontally transferred sequences are expressed and RNA edited in any of the endoparasites, we performed RT-PCR from *Rafflesia cantleyi *floral RNA and compared the *atp1 *cDNA sequence to the DNA sequence from the same individual. Figure 4 shows that not only was cDNA obtained from the *Rafflesia *RNA, suggesting that *atp1 *is expressed, it appears that the RNA undergoes editing because of the observed T at position 931 in the cDNA relative to C in the same position of the DNA sequence. This result is consistent with a C to U edit at this site in the transcript. The fact that the *atp1 *transcript is edited suggests that it is transcribed in the mitochondrion of this species. This edit site is at a nonsynonymous, first codon position that results in the encoding of serine rather than proline. This RNA edit site has not been reported for other species [34].

**Figure 5:**
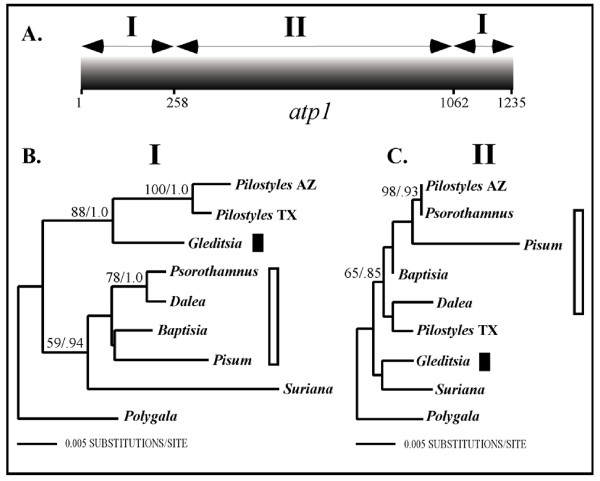
**Chimeric nature of *atp1 *in *Pilostyles thurberi***. A. Spatial delimitation of regions I and II of *atp1 *based on gene conversion analyses of sequence variation in *Pilostyles *and various Fabalean taxa. A region of 804 bp in *Pilostyles *(AZ) (region II) was inferred to be the result of gene conversion by an *atp1 *sequence from its host, *Psorothamnus *(*P *< 0.05). B. Single most likely tree obtained in phylogenetic analyses of regions I (-lnL = 883.42). C. Single most likely tree obtained in phylogenetic analyses of regions II (-lnL = 1383.82). Filled bar indicates caesalpinioid legumes and unfilled bar indicates faboid legumes. BP/PP values are shown above all nodes with values >50/0.8.

When comparing the *atp1 *sequences of two accessions of *Pilostyles thurberi *(one from Arizona [AZ] and one from Texas [TX]), we noted that the two sequences differed at ten sites. This is a surprisingly large number of differences considering these are accessions of the same species and that the mutation rate of mitochondrial DNA in plants is typically very low [35] (although see [36] and [37] for two rare exceptions). Eight of the differences were restricted to the central region of the gene. In contrast, comparisons of *atp1 *from *Pilostyles *(AZ) to the host *Psorothamnus *individual it was collected from, revealed that the two species were identical for 804 bp in the central region of the gene, but have a disproportionate 15 differences at the 5' and 3' ends. Because the *Pilostyles *(AZ) sequence was obtained in multiple PCR amplifications from independent samples, the result is not likely explained by PCR-mediated recombination. Instead, we hypothesized that *Pilostyles *(AZ) *atp1 *may represent a chimeric xenolog that is the result of multiple historical horizontal gene transfer events from its hosts. To investigate this possibility, we performed gene conversion analyses that detected an 804 bp region of *Pilostyles *(AZ) (region II; Fig. 5A) that appears to have been converted by its current host sequence (*P *< 0.05). Separate phylogenetic analyses of the two regions including multiple members of Fabales reinforced the hypothesis of a different history for these two regions as well. Regions I of *atp1 *show that the two *Pilostyles *sequences are nested within Fabales and are most closely related to the caesalpinioid legume, *Gleditsia *(Fig. 5B), whereas the putatively, recently converted region II shows that *Pilostyles *(AZ) is most closely related to its current faboid host, *Psorothamnus *(Fig. 5C). Interestingly, the region II tree also suggests that *Pilostyles *(TX) may have independently horizontally acquired *atp1 *multiple times as well, perhaps recently from its modern host, a species of *Dalea *(Fig. 5C). An S-H test suggested that the tree estimated from the putatively converted region (region II; Fig. 5) is statistically incongruent with the data of the adjacent 5' and 3' regions (regions I; Fig. 5) (*P *= 0.002) and vice versa (*P *= 0.009).

### *coxI *intron acquisition in parasitic plants

**Figure 6:**
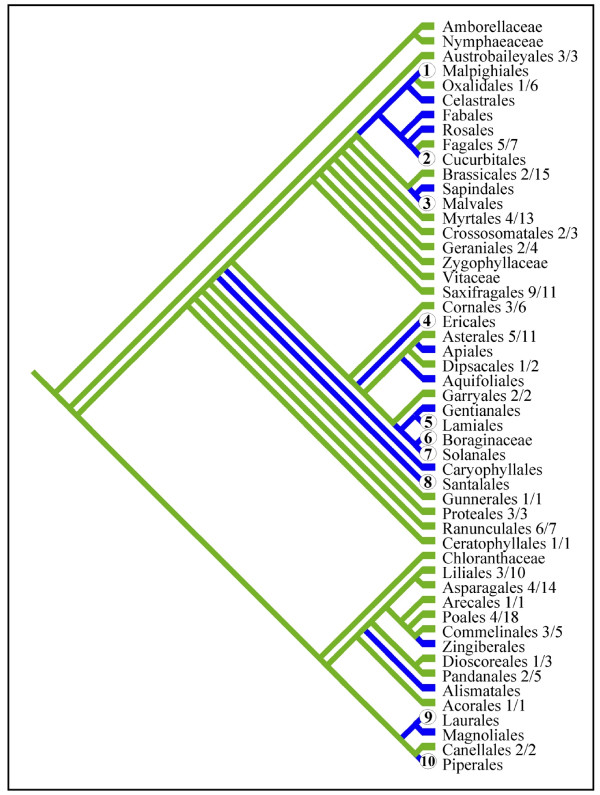
**Ordinal level analysis of *coxI *intron presence among angiosperms**. Character state tracing of *coxI *intron presence (shown in blue) among 45 orders of angiosperms. Numbers listed next to intron-negative orders show the number of families sampled for the *coxI *intron out of the total currently included within each order. Parasitism is inferred to have evolved 10 times on branches that also are inferred to have the intron (shown by numbers within circles). Only Krameriaceae and Cynomoriaceae do not appear to be associated with intron containing lineages. The probability of 10 origins of parasitism and zero losses of parasitism on branches that have the intron is < 0.001.

A final striking and unexpected observation of mtDNA sequence variation is that the mt *coxI *gene in nearly every parasitic lineage has been invaded by a group I intron (all lineages with the *coxI *intron are marked by  or ● in Fig. 1). The only sampled parasitic plants lacking introns are *Krameria *and *Schoepfia*, both hemiparasites; all other sampled hemiparasites and all holoparasites possess the intron. Multiple independent invasions and rampant horizontal transfer of this intron in angiosperms has been previously reported [28]; however, no correlation to life history, biogeography or phylogeny was found. Yet, it appears that there is a correlation between the evolution of parasitism and the presence of the *coxI *intron. The probability of observing 10 gains and 0 losses of parasitism on branches with the *coxI *intron as reconstructed on the tree shown in Fig. 1 is very low (*P *< 0.001). However, to avoid potential bias due to our exhaustive sampling of parasite lineages, we investigated this question further at the ordinal level. Based on our data shown in Fig. 1 and literature reports [28], more than 350 species of plants from all monophyletic orders of flowering plants (sensu [13]) have been surveyed for the intron. Using this innformation, we assigned intron presence or absence to every order, as well as 6 families that are not currently placed in an order (Amborellaceae, Nymphaeaceae, Chloranthaceae, Vitaceae, Zygophyllaceae, and Boraginaceae) (Fig. 6). Out of the 24 orders that we have scored as intron negative, 16 have had at least half of the included families sampled for the *coxI *intron (Fig. 6). Assuming this tree representing relationships among all orders of angiosperms, the probability of observing 10 gains and 0 losses of parasitism is < 0.001. However, because we have sampled less than half of the included families from Oxalidales, Brassicales, Myrtales, Asterales, Liliales, Asparagales, Poales, and Pandanales, we investigated the effect of re-scoring these orders as intron positive on the observed correlation. Even in this case, the probability of observing 10 gains and 0 losses of parasitism is < 0.01. The results are quantitatively similar regardless if we use ACCTRAN or DELTRAN to resolve equivocal ancestral states or if we resolve the polytomous relationships presented in [13] differently from what is shown.

**Figure 7:**
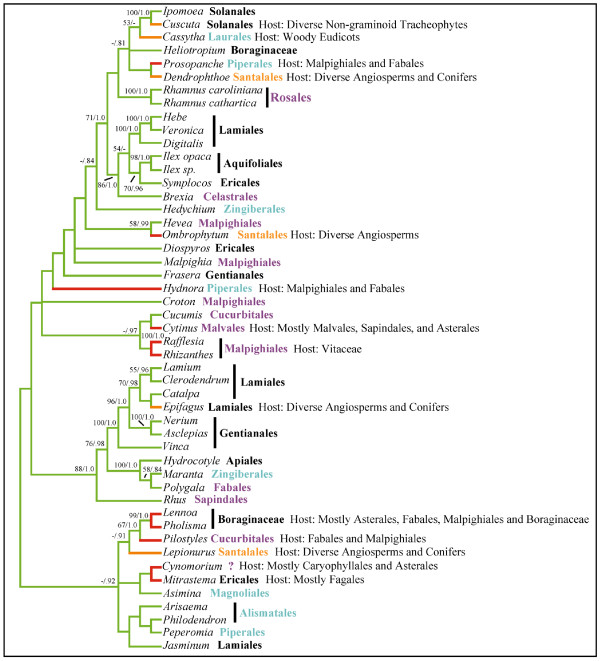
***coxI *intron phylogeny in flowering plants**. Phylogenetic relationships among 49 angiosperm *coxI *intron DNA sequences inferred using ML (-lnL = 5938.60). Lineages shown in green are non-parasitic. Lineages in orange are mostly hemiparasitic, while those in red are holoparasitic. Bootstrap support values >50 and posterior probabilities > 0.8 are shown before and after the "/" respectively. Ordinal (or familial, if currently unplaced to order) classification is shown next to each taxon. Basal angiosperms and monocot orders are labeled in blue, rosid orders are labeled in purple, asterid orders are labeled in black, and Santalales are shown in yellow. General host plant preference for each parasitic plant lineage is shown next to each parasite (based on ref. # 3 and personal observations by TJB, JRM, and CWD). Overall phylogenetic relationships of the intron sequences are highly discordant with angiosperm phylogeny. Horizontal acquisition of the intron in parasites from their hosts does not seem likely because in no case is a highly supported relationship found between a parasite and any of its host lineages. Vertical acquisition of the intron in *Cuscuta *and *Epifagus *is supported by this tree because of the highly supported relationship found between these parasites and their close relatives.

One hypothesis for the apparent association of parasitism and the *coxI *intron is that the parasites have horizontally acquired the intron from their current hosts. This hypothesis is not supported by our data because in cases where the parasite is highly host-specific, the host lineages do not have an intron. For example, *Rafflesia *and *Rhizanthes *only parasitize *Tetrastigma *(Vitaceae), yet this genus (and other sampled members of the family) does not have an intron, and there is no evidence that it ever had one, as indicated by the lack of intron co-conversion sites [28]. In other cases, such as *Cuscuta *and Orobanchaceae, the parasite introns are closely related to intron sequences of close non-parasitic relatives and thus appear to have been transmitted vertically from non-parasitic ancestors (Fig. 7). Overall, the intron phylogeny is highly discordant with angiosperm phylogeny as indicated by a significant S-H test comparing the optimal tree to one that constrains asterids rosids, orders, and families of plants to be monophyletic (*P *< 0.05). This results corroborates earlier findings [28] of widespread HGT of this mobile sequence (Fig. 7).

## Discussion

### Phylogenetic aspects of parasitism

The pattern of evolution of haustorial parasitism in angiosperms is striking in several regards. First, it appears that parasitism has evolved repeatedly in many major groups of flowering plants from magnoliids to derived eudicot lineages, and most of these lineages (8 of 11–13) now consist entirely of nonphotosynthetic parasites. However, monocots, campanulids, and caryophyllids (ca. 22%, 12%, and 7% of angiosperm diversity, respectively) have never evolved parasitism or else retain no extant parasitic representatives. In contrast, within lamiids alone (ca. 12% of angiosperm diversity), parasitism has independently evolved 3 times including both shoot (*Cuscuta*) and root (Orobanchaceae and Lennoaceae) parasites, although this may not be statistically different from zero. Why some lineages have a propensity to become parasitic is not clear; however, the strong correlation of the distribution of the *coxI *intron (which, at the ordinal level, is also rare in monocot and campanulid orders, but rich throughout rosid and lamiid orders) with the independent origins of parasitism (Fig. 1 and 6) is tantalizing and will be discussed below. Second, the highly specialized trait of endoparasitism [3], in which most of the vegetative portion of the parasite lives inside of its host with emergence occurring during sexual reproduction, has evolved independently in at least 4 lineages, Mitrastemonaceae, Cytinaceae, Rafflesiaceae, and Apodanthaceae. Furthermore, although not studied here, *Arceuthobium *(Santalales) is endoparasitic [3]. Evolutionary convergence of the endoparasitic lifestyle in one-third of the parasitic angiosperm lineages and only in the most derived species of Santalales suggests this may be a common adaptive peak of parasite-host relationships. As in animals, a selective advantage of the endoparasitic mode for plants may be to avoid predators (herbivores) and live in a homeostatic environment [1]. Third, this ordinal level-placement of all parasitic angiosperms (except *Cynomorium*), corroborates other recent studies and together resolve many long-standing taxonomic questions. Thus, revisions of existing classifications are needed to include many of these parasites in orders they have not been placed in previously. This is particularly true of Rafflesiaceae, Mitrastemonaceae, Cytinaceae, and Apodanthaceae. Further work is required to refine the positions of most parasitic plants within their orders. However, use of mtDNA will require cognizance of the possibilities of HGT from host-to-parasite [21] and also from parasite-to-host [25,27]. Surprisingly, in spite of the conflicting nature of *atp1 *relative to *coxI *and *matR *in the endoparasites Rafflesiaceae and Mitrastemonaceae, confident placements were obtained in the combined analyses. In contrast, the position of *Cynomorium *is obscured by the conflicting phylogenetic positions implied by *matR *(Saxifragales) and *coxI *+ *atp1 *(Sapindales). This conflict could suggest possible horizontal transfer (HGT) of *matR *or both *coxI *and *atp1 *in *Cynomorium*; however, neither Sapindalean nor Saxifragalean hosts have been described in the literature to our knowledge. A placement of *Cynomorium *with Saxifragales has been suggested by 18S as well as *matR *[12].

### Multiple Horizontal Gene Transfers of *atp1*

One of the major predicted consequences of long-term parasitic interactions is that genetic transfer will occur between host and parasite [1,2,38]. Although evidence to support this hypothesis has been scarce in eukaryotes, the phylogenetic evidence we present from *atp1 *of endoparasitic plants is consistent with this prediction. While HGT is plausible from host-to-parasite, it is not clear that such transfers should be advantageous to recipients. One possibility in parasites is that if their native mt loci were degenerate but could have been replaced by a highly conserved copy, then transfer should be strongly selected for because it would help ensure efficient metabolism in these extreme holoparasites. Yet, there is no statistical evidence for this hypothesis in the chimeric *atp1 *sequences of *Pilostyles *because the retained portions of the presumably older caesalpiniod xenolog (regions I) are not divergent at nonsynonymous sites relative to photosynthetic plants. Regardless of any potential selective advantage of HGT, it appears that *atp1 *is mobile because of the multiple transfers that we and others have reported [19,23,27]. Because *atp1 *appears to be located near sites of recombination in some plants [39], its mobility may be facilitated. Further support for HGT in parasitic plants may come from surveys of other genes, particularly from the nuclear genome of these parasites.

The close phylogenetic relationships of *atp1 *between endoparasites and their hosts, the statistically significant phylogenetic conflict of *atp1 *relative to *matR *+ *coxI*, and the differential evolutionary dynamics of *atp1 *relative to *coxI *and *matR*, suggest that *atp1 *has been acquired horizontally in *Rafflesia *+ *Rhizanthes*, *Pilostyles*, and *Mitrastema *from their respective host lineages. The possibility of HGT of plant mtDNA, including *atp1*, has been raised in other angiosperms [19-23] and seems a likely explanation for our results for several reasons. First, the horizontal transfer of macromolecules, including RNA, from host plant to parasitic plant has been shown to occur experimentally [40,41]. Second, these parasites (*Rafflesia*, *Rhizanthes*, *Pilostyles*, and *Mitrastema*) are wholly endoparasitic angiosperms; they vegetatively grow completely embedded within their hosts to enhance the acquisition of water, nutrients, and complex macromolecules. Third, because reproductive tissues arise from the endophytic vegetative tissue in these species, cells that carry horizontally acquired host DNA are likely to give rise to reproductive meristems and transmit the new DNA to future generations of the parasitic plant. Future studies should aim to determine if a native copy of *atp1 *still exists in these parasites and characterize the genomic location and flanking sequence of putatively transferred sequences.

### *coxI *intron invasion and genomic chimerism

Although there have been multiple horizontal acquisitions of the homing *coxI *intron throughout angiosperm history [28], it is clear that this invasive sequence is more prone to invade some angiosperm lineages than others (eg. lamiids and parasites). While the source of the intron is unclear in the parasites (and angiosperms in general [28]), the acquisition of foreign DNA has been predicted to be a key event in the evolution of parasitic angiosperms [38], and this intron could represent a marker of a genomically more widespread historical transformation. This is a particularly interesting possibility because the *coxI *intron in angiosperms is most closely related to those known from fungi and the evolution of haustorial parasitism has been hypothesized to have occurred via a mycoheterotrophic antecedent relationship [3]. Recently, genomic comparisons have revealed that the shift to parasitism in nematodes may have been facilitated by the recent acquisition of foreign DNA from bacteria [42]. Whether the acquisition of foreign DNA was a key step in the evolution of parasitism in plants awaits genomic studies of these parasites.

## Conclusion

Genomic chimerism among angiosperms is probable given that there have been multiple origins of parasitism throughout flowering plant history and that HGT is possible from host to parasite [21] and vice versa [27]. Although some parasitic lineages are highly host specific, many others have a broad range of potential host species (Fig 7), and host shifting is to be expected through the history of individual parasite lineages [1-3]. Therefore, even if HGT between plant parasites and their hosts is very rare, through time this could result in plant genomes that are complex chimeras of horizontally as well as vertically acquired sequences.

## Methods

### Taxon sampling

We sampled representatives of at least one family from 44 of 45 orders from the recent ordinal classification of angiosperms [13]. In total, 102 seed plant species from 92 angiosperm families were represented in addition to every major parasitic plant group [3,5]. Three gymnosperms, *Pinus*, *Ginkgo*, and *Zamia*, were included as outgroups to root phylogenetic estimates. Although we attempted to use the same DNA for all gene isolation, some composite taxa were used in our analyses (see Additional file 1). In the case of one of our samples of Malpighiales, *Euphorbia milli *was used for *matR *and *Croton alabamensis *for *coxI *and *atp1*. In the case of Malvales, *Alcea rosea *was used for *matR *and *Althaea officinalis *for *coxI *and *atp1*. In the rest of the cases, we isolated the 3 genes from the same DNA or different species of the same genus (see Additional file 1).

### Molecular methods

General molecular methods, including DNA extraction, PCR, and DNA sequencing were performed as previously described [11,43]. The basic RNA extraction and RT-PCR procedures followed published methods [44]. Great care was taken to limit the possibility of host plant or other contamination of the parasitic plant DNA samples, including careful dissection of tissues distal to the host-parasite interface as well using multiple independent isolations from two laboratories and/or related species or genera. Furthermore, we also sampled the host plant individuals of the holoparasites, *Rafflesia pricei *(*Tetrastigma diepenhorstii*), *Mitrastema yamamotoi *(*Quercus subsericea*), and *Pilostyles thurberi *(AZ) (*Psorothamnus emoryi*) so they could be directly compared. This is a critical aspect of our study and is absolutely necessary in order to discriminate between putative cases of horizontal gene transfer and contamination or the presence of phloem-mobile nucleic acids taken up by parasites. Please see Additional files 1 and 2 for the voucher numbers and GenBank accession numbers for all of the sequences included in this study. In total, 188 new mtDNA sequences were generated.

### Phylogenetic analyses

ClustalX [45] was used to produce preliminary sequence alignments followed by minor manual adjustments. Regions of uncertain alignment from all three genes, *coxI *intron sequences [28], and known RNA editing sites for *atp1 *and *coxI *were excluded prior to analysis [46]. Modeltest v3.06 [47] was used to determine the best-fit model of nucleotide substitution for each data set analyzed; these models were implemented during maximum likelihood (ML) analyses with PAUP*4.0 [48]. ML heuristic searches used 10 random addition sequences and TBR swapping. Support values could not be determined using ML because of the large computation time required, so bootstrap support (BP) values were obtained using GARLI [49] from 100 replicates using an automated stopping criterion set to 5,000 generations. Bayesian analyses were performed using MrBayes v3.0b4 [50]. Five chains were simultaneously run for one million generations and these were sampled every 100 generations. The first 10,000 generations were discarded as the "burn-in" period because of convergence lnL after this point and posterior probabilities (PP) for individual clades were then obtained from the remaining samples. Corrected pairwise divergences were calculated using the optimal models of nucleotide substitution determined by Modeltest for each gene separately. The S-H test [51] was used to test for statistically significant differences among competing topological hypotheses. Putative gene conversion events were identified using GeneConv [52]. The concentrated changes test [53] was used to test for non-random association between parasitism and the presence of the *coxI *intron by first tracing the intron distribution onto the tree in Fig. 1 or Fig. 6. We tested the null hypothesis that gains and losses of parasitism are randomly distributed over the angiosperm phylogenetic tree. Probabilities for the 10 gains and 0 losses of parasitism occurring in lineages that possess the *coxI *intron were then determined by 1,000 simulations.

## Authors' contributions

TJB conceived the study, gathered gene and intron sequences, contributed to the specimen collection, performed data analyses and wrote and edited the manuscript.

JRM gathered gene and intron sequences, contributed to the specimen collection, performed data analyses, and contributed to the data interpretations and writing of the manuscript.

S-HL gathered gene sequences and contributed to the specimen collection

GC gathered most of the *atp1 *gene sequences for the study

HBC surveyed the *coxI *intron distribution in parasitic and nonparasitic plants, and initiated the *coxI *sequencing efforts

NY conceived of the study, contributed to the specimen collection and *coxI *intron survey, and helped initiate the sequencing efforts.

CWD conceived of the study, developed the intron tests, and contributed to the specimen collection, data analysis and interpretations, and writing and editing of the manuscript.

All authors read and approved the final manuscript.

## Supplementary Material

Additional File 1 Table of voucher numbers and GenBank accession numbers. Click here for file

Additional File 2 Table of voucher numbers and GenBank accession numbers. Click here for file
