# Supplementary material for: Mitochondrial DNA suggests at least 11 origins of parasitism in angiosperms and reveals genomic chimerism in parasitic plants
Source: BMC Evol Biol. 2007 Dec 21;7:248. doi: 10.1186/1471-2148-7-248 (PMC2234419; doi:10.1186/1471-2148-7-248)
Supplement: Additional File 1 — Table of voucher numbers and GenBank accession numbers. [file 1471-2148-7-248-S1.pdf]

Table 1. List of species sampled, voucher numbers with herbarium acronyms, and GenBank accession numbers. Sequences obtained from GenBank and DNA obtained from other published studies do not list voucher information.

Species Name	Voucher Number	*matR*	Species Name	Voucher Number	*coxI*	Species Name	Voucher Number	*atp1*
Ailanthus altissima	TJB 344 (WMU)	EU281096	*Ailanthus altissima*	TJB 344 (WMU)	EU281024	*Ailanthus altissima*	TJB 344 (WMU)	EU280952
Akebia quinata	N/A	AF197810	*Akebia quinata*	N/A	AY009429	*Akebia quinata*	N/A	AY009406
Alcea rosea	N/A	AY453094	*Althaea officinalis*	GRIN 440121(no voucher)	EU281042	*Althaea officinalis*	GRIN 440121(no voucher)	EU280970
Alstroemeria hybrid	N/A	AY453076	*Alstroemeria hybrid*	TJB 347 (PAC)	EU281072	*Alstroemeria hybrid*	TJB 347 (PAC)	EU280997
Amborella trichopoda	N/A	AF197813	*Amborella trichopoda*	N/A	AY009430	*Amborella trichopoda*	N/A	AY009407
Amoreuxia wrightii	DHG 1346 (TEX)	EU281098	*Amoreuxia wrightii*	DHG 1346 (TEX)	EU281028	*Amoreuxia wrightii*	DHG 1346 (TEX)	EU280957
Arabidopsis thaliana	N/A	NC 001284	*Arabidopsis thaliana*	N/A	Y08502	*Arabidopsis thaliana*	N/A	Y08502
Aralia spinosa	CWD 97.807 (PAC)	EU281115	*Aralia spinosa*	CWD 97.807 (PAC)	EU281059	*Aralia spinosa*	CWD 97.807 (PAC)	EU280985
Arisaema triphyllum	TJB351 (WMU)	EU281121	*Arisaema triphyllum*	N/A	AY009454	*Arisaema triphyllum*	N/A	AY009426
Asclepias tuberosa	N/A	AY453103	*Asclepias tuberosa*	KRAL 70275 (BRIT)	EU281054	*Asclepias viridis*	KRAL 80538 (BRIT)	EU280981
Asimina triloba	N/A	AF197765	*Asimina triloba*	N/A	AY009433	*Asimina triloba*	N/A	AY009409
Austrobaileya scandens	N/A	AF197742	*Austrobaileya scandens*	N/A	AY009434	*Austrobaileya scandens*	N/A	AY009410
Begonia sp.	N/A	AY453119	*Begonia sp.*	TJB 346 (PAC)	EU281027	*Begonia sp.*	TJB 346 (PAC)	EU280955
Betula pedula	N/A	AY453121	*Betula papyrifera*	N/A	U77620	*Betula nigra*	CWD97.558 (PAC)	EU280956
Borago officinalis	N/A	EU281099	*Borago officinalis*	N/A	EU281029	*Borago officinalis*	N/A	EU280958
Bursera simarouba	DHG1365 (TEX)	EU281100	*Bursera simarouba*	DHG1365 (TEX)	EU281030	*Bursera simarouba*	DHG1365 (TEX)	EU280959
Cabomba sp.	N/A	AF197729	*Cabomba caroliniana*	N/A	AY009435	*Cabomba sp.*	N/A	AF197641
Canella winterana	N/A	AF197757	*Canella winterana*	N/A	AY009437	*Canella winterana*	N/A	AY009412
Canna indica	SKJ29Jan99 (PAC)	EU281101	*Canna warscewiczii*	CWD 94.90 (PAC)	EU281031	*Canna indica*	SKJ29Jan99 (PAC)	EU280960
Cassytha filiformis	TJB340 (WMU)	EU281123	*Cassytha filiformis*	CWD97.731 (PAC)	EU281076	*Cassytha filiformis*	CWD97.731 (PAC)	EU281002
Ceratophyllum demersum	N/A	AF197730	*Ceratophyllum demersum*	N/A	AY009438	*Ceratophyllum demersum*	N/A	AY009413
Chloranthus multistachys	N/A	AF197753	*Chloranthus spicatus*	N/A	AY009439	*Chloranthus multistachys*	N/A	AF197665
Cinnamomum camphora	N/A	AF197797	*Cinnamomum zeylanicum*	N/A	AY009440	*Cinnamomum zeylanicum*	N/A	AY009415
Clethra arborea	N/A	AF420996	*Clethra alnifolia*	CWD 96.319 (PAC)	EU281032	*Clethra arborea*	N/A	AF420919
Cuscuta japonica	no voucher	EU281124	*Cuscuta japonica*	no voucher	EU281077	*Cuscuta japonica*	no voucher	EU281003
Cynomorium coccineum	Hoder 18/Apr/1996 (PAC)	EU281095	*Cynomorium coccineum*	Hoder 18/Apr/1996 (PAC)	EU281023	*Cynomorium coccineum*	Hoder 18/Apr/1996 (PAC)	EU280951
Cytinus ruber	N/A	EU281094	*Cytinus ruber*	KES2738 (BOL)	EU281022	*Cytinus ruber*	KES2738 (BOL)	EU280950
Dendrophthoe pentandra	N/A	AY453120	*Dendrophthoe pentandra*	SNP15593 (SNP)	EU281073	*Dendrophthoe pentandra*	SNP15593 (SNP)	EU280999
Dicentra sp.	N/A	AF197796	*Dicentra spectabilis*	TJB404 (WMU)	EU281034	*Dicentra sp.*	N/A	AF197649
Digitalis purpurea	CWD93.41 (PAC)	EU281103	*Digitalis purpurea*	N/A	AJ223415	*Digitalis purpurea*	CWD93.41 (PAC)	EU280962
Dioscorea sp.	N/A	AF197737	*Dioscorea macrostachya*	N/A	AY009442	*Dioscorea macrostachya*	N/A	AY009417
Dipsacus fullonum	N/A	AY453093	*Dipsacus fullonum*	SKJ8Jan99 (PAC)	EU281035	*Dipsacus fullonum*	SKJ8Jan99 (PAC)	EU280963
Drimys winteri	N/A	AF197781	*Drimys winteri*	N/A	AY009443	*Drimys winteri*	N/A	AY009418
Echinocytis lobata	JRM 98.0805 (PAC)	EU281102	*Echinocytis lobata*	JRM 98.0805 (PAC)	EU281033	*Echinocytis lobata*	JRM 98.0805 (PAC)	EU280961
Epidendrum sp.	CWD 94.10 (PAC)	EU281113	*Epidendrum sp.*	CWD 94.10 (PAC)	EU281056	*Epidendrum sp.*	CWD 94.10 (PAC)	EU280982
Epifagus virginiana	CWD 90.127 (PAC)	EU281125	*Epifagus virginiana*	CWD 90.127 (PAC)	EU281078	*Epifagus virginiana*	CWD 90.127 (PAC)	EU281004
Eucommia ulmoides	CWD 97.1009 (PAC)	EU281104	*Eucommia ulmoides*	CWD 97.1009 (PAC)	EU281036	*Eucommia ulmoides*	CWD 97.1009 (PAC)	EU280964
Euonymus fortunei	N/A	AY453104	*Euonymus americanus*	CWD97.538 (PAC)	EU281074	*Euonymus americanus*	CWD97.538 (PAC)	EU281000
Euphorbia millii	TJB384 (WMU)	EU281105	*Croton alabamensis*	CWD96.303 (PAC)	EU281037	*Croton alabamensis*	CWD96.303 (PAC)	EU280965
Eupomatia bennettii	N/A	AF197772	*Eupomatia laurina*	N/A	AY009444	*Eupomatia bennettii*	N/A	AF197692
Fagus sylvatica	N/A	AY453092	*Fagus grandifolia*	CWD 97.519 (PAC)	EU281039	*Fagus grandifolia*	CWD 97.519 (PAC)	EU280967
Frasera caroliniensis	CWD 97.537 (PAC)	EU281106	*Frasera caroliniensis*	CWD 97.537 (PAC)	EU281038	*Frasera caroliniensis*	CWD 97.537 (PAC)	EU280966
Garrya elliptica	N/A	AY453095	*Garrya elliptica*	CWD 97.1050 (PAC)	EU281041	*Garrya elliptica*	CWD 97.1050 (PAC)	EU280969
Ginkgo biloba	N/A	AF197722	*Ginkgo biloba*	N/A	AF020565	*Ginkgo biloba*	N/A	AF209110
Griselinia racemosa	N/A	AY453096	*Griselinia racemosa*	N/A	EU281087	*Griselinia racemosa*	N/A	EU281012
Gunnera sp.	CWD 94.84 (PAC)	EU281129	*Gunnera sp.*	CWD 94.84 (PAC)	EU281086	*Gunnera sp.*	CWD 94.84 (PAC)	EU281011
Helianthemum sp.	no voucher	EU281108	*Helianthemum sp.*	no voucher	EU281044	*Helianthemum sp.*	no voucher	EU280972
Helianthus annuus	N/A	AY453114	*Helianthus annuus*	KES5/20/1992 (PAC)	EU281045	*Helianthus annuus*	N/A	X52838
Humulus lupulus	CWD 97.1010 (PAC)	EU281110	*Humulus lupulus*	CWD 97.1010 (PAC)	EU281047	*Humulus lupulus*	CWD 97.1010 (PAC)	EU280974
Hydnora africana	N/A	AF503358	*Hydnora africana*	N/A	EU281079	*Hydnora africana*	N/A	EU281005
Hydrangea macrophylla	N/A	AY453091	*Hydrangea seemanii*	CWD 94.85 (PAC)	EU281048	*Hydrangea seemanii*	CWD 94.85 (PAC)	EU280975
Ilex aquifolium	N/A	AY453090	*Ilex opaca*	CWD97.656 (PAC)	EU281049	*Ilex opaca*	CWD97.656 (PAC)	EU280976
Illicium floridanum	N/A	AF197740	*Illicium lanceolatum*	N/A	AY009445	*Illicium lanceolatum*	N/A	AF209101
Ipomoea alba	TR020 (WMU)	EU281111	*Ipomoea coccinea x quamoclit*	JRM97.GRHS1083 (PAC)	EU281050	*Ipomoea coccinea x quamoclit*	JRM97.GRHS1083 (PAC)	EU280977
Iris pseudacorus	PJL 1128 (WMU)	EU281097	*Iris pseudacorus*	CWD 93.11 (PAC)	EU281026	*Iris pseudacorus*	CWD 93.11 (PAC)	EU280954
Jasminum floridum	CWD90.69 (PAC)	EU281112	*Jasminum floridum*	CWD90.69 (PAC)	EU281051	*Jasminum floridum*	CWD90.69 (PAC)	EU280978
Kallstroemia parviflora	N/A	AY453122	*Kallstroemia parviflora*	DHG 1363 (TEX)	EU281043	*Kallstroemia parviflora*	DHG 1363 (TEX)	EU280971
Krameria lanceolata	N/A	AY453089	*Krameria lanceolata*	DHG 1362 (TEX)	EU281052	*Krameria lanceolata*	DHG 1362 (TEX)	EU280979
Lennoa madreporoides	OY6 (PAC)	EU281126	*Lennoa madreporoides*	OY6 (PAC)	EU281080	*Lennoa madreporoides*	OY6 (PAC)	EU281006
Liquidambar styraciflua	CWD94.115 (PAC)	EU281130	*Liquidambar styraciflua*	CWD94.115 (PAC)	EU281088	*Liquidambar styraciflua*	CWD94.115 (PAC)	EU281013
Magnolia tripetala	N/A	AF 197770	*Magnolia grandiflora*	N/A	AF020568	*Magnolia grandiflora*	N/A	AF209100
Melianthus major	N/A	AY453087	*Melianthus major*	TJB357 (WMU)	EU281085	*Melianthus major*	TJB357 (WMU)	EU281010
Mirabilis jalapa	N/A	AY453086	*Mirabilis jalapa*	DHG 1341 (TEX)	EU281053	*Mirabilis jalapa*	DHG 1341 (TEX)	EU280980
Mitrastema yamamotoi	N/A	AY453116	*Mitrastema yamamotoi*	CWD 99.11 (SNP)	EU281021	*Mitrastema yamamotoi*	CWD 99.11 (SNP)	EU280949
Myrtus communis	CWD 97.1012 (PAC)	EU281109	*Myrtus communis*	CWD 97.1012 (PAC)	EU281046	*Myrtus communis*	CWD 97.1012 (PAC)	EU280973
Nelumbo nucifera	N/A	AF197795	*Nelumbo lutea*	N/A	AY009447	*Nelumbo lutea*	N/A	AY009420
Nicotiana sylvestris	N/A	AY453113	*Nicotiana tabacum*	CWD 30 (PAC)	EU281055	*Nicotiana plumbaginifolia*	N/A	X07745
Nymphaea sp.	N/A	AF197727	*Nymphaea odorata*	N/A	AF020570	*Nymphaea odorata*	N/A	AF209102
Oenothera biennis	N/A	AY453083	*Oenothera biennis*	N/A	AF020571	*Oenothera biennis*	N/A	X04023
Ombrophytum subterraneum	CWD 94.17 (PAC)	EU281127	*Ombrophytum subterraneum*	CWD 94.17 (PAC)	EU281081	*Ombrophytum subterraneum*	CWD 94.17 (PAC)	EU281007
Oryza sativa	N/A	AB 076665	*Oryza sativa*	N/A	X15990	*Oryza sativa*	N/A	AB076666
Oxalis corniculata	N/A	AY453111	*Oxalis stricta*	KES20May92 (PAC)	EU281057	*Oxalis stricta*	KES20May92 (PAC)	EU280983
Paeonia lactiflora	TJB360 (WMU)	EU281114	*Paeonia hybrid*	CWD 96.3 (PAC)	EU281058	*Paeonia hybrid*	CWD 96.3 (PAC)	EU280984
Pandanus sp.	SKJ8Jan99 (PAC)	EU281107	*Pandanus sp.*	SKJ8Jan99 (PAC)	EU281040	*Pandanus sp.*	SKJ8Jan99 (PAC)	EU280968
Phoenix theophrastii	CWD 94.104 (PAC)	EU281116	*Phoenix theophrastii*	CWD 94.104 (PAC)	EU281060	*Phoenix reclinata*	N/A	PRU58831
Pholisma arenarium	RAS120 (PAC)	EU281128	*Pholisma arenarium*	RAS120 (PAC)	EU281083	*Pholisma arenarium*	RAS120 (PAC)	EU281008
Pilostyles thurberi (AZ)	CWD2004.0A (PAC)	EU281132	*Pilostyles thurberi* (AZ)	CWD2004.0A (PAC)	EU281092	*Pilostyles thurberi* (AZ)	CWD2004.0A (PAC)	EU281017
Pilostyles thurberi (TX)	Turner 99-270 (TEX)	EU281093	*Pilostyles thurberi* (TX)	Turner 99-270 (TEX)	EU281018	*Pilostyles thurberi* (TX)	Turner 99-270 (TEX)	EU280946
Pinus sp.	N/A	AF197723	*Pinus strobus*	N/A	AF020574	*Pinus strobus*	N/A	AF209108
Piper betle	N/A	AF197750	*Piper bicolor*	N/A	AY009448	*Piper betle*	N/A	AF197630
Pisum sativum	N/A	AY453078	*Pisum sativum*	N/A	X14409	*Pisum sativum*	N/A	D14698
Platanus occidentalis	N/A	AF197793	*Platanus occidentalis*	N/A	AY009450	*Platanus occidentalis*	N/A	AY009423
Polygala paucifolia	N/A	AY453080	*Polygala sanguinea*	CWD98.0804 (PAC)	EU281061	*Polygala sanguinea*	CWD98.0804 (PAC)	EU280986
Pontederia lanceolata	CWD 97.1045 (PAC)	EU281117	*Pontederia lanceolata*	CWD 97.1045 (PAC)	EU281062	*Pontederia lanceolata*	CWD 97.1045 (PAC)	EU280987
Prosopanche americana	N/A	AF503359	*Prosopanche americana*	OY26 (PAC)	EU281082	*Prosopanche americana*	N/A	AF503357
Psorothamnus emoryi	CWD2004.0B (PAC)	EU281132	*Psorothamnus emoryi*	CWD2004.0B (PAC)	EU281089	*Psorothamnus emoryi*	CWD2004.0B (PAC)	EU281014
Quercus subsericea	SNP10161 (SNP)	EU281131	*Quercus subsericea*	SNP10161 (SNP)	EU281090	*Quercus subsericea*	SNP10161 (SNP)	EU281015
Rafflesia keithii	N/A	AY453074	*Rafflesia pricei*	CWD 99.01 (SNP)	EU281020	*Rafflesia pricei*	CWD 99.01 (SNP)	EU280948
Rhamnus cathartica	TJB392 (WMU)	EU281118	*Rhamnus caroliniana*	CWD96.315 (PAC)	EU281063	*Rhamnus caroliniana*	CWD96.315 (PAC)	EU280988
Rhizanthes lowii	N/A	AY453073	*Rhizanthes lowii*	SNP14705 (SNP)	EU281019	*Rhizanthes lowii*	SNP14705 (SNP)	EU280947
Rhus typhina	N/A	AY453075	*Rhus glabra*	CWD 97.522 (PAC)	EU281065	*Rhus glabra*	CWD 97.522 (PAC)	EU280990
Salix babylonica	N/A	AY453072	*Salix nigra*	CWD 96.322 (PAC)	EU281084	*Salix nigra*	CWD 96.322 (PAC)	EU281009
Saururus chinensis	N/A	AF332106	*Saururus chinensis*	N/A	AY009452	*Saururus chinensis*	N/A	AY009424
Scaevola aemula	N/A	AY453118	*Scaevola plumeri*	CWD 97.720 (PAC)	EU281064	*Scaevola plumeri*	CWD 97.720 (PAC)	EU280989
Schisandra sphenanthera	N/A	AF197739	*Schisandra henryi*	N/A	AY009453	*Schisandra henryi*	N/A	AY009425
Schoepfia	JKP4OCT96 (PAC)	EU281119	*Schoepfia*	JKP4OCT96 (PAC)	EU281066	*Schoepfia*	JKP4OCT96 (PAC)	EU280991
Smilax sp.	CWD 97.531 (PAC)	EU281120	*Smilax sp.*	CWD 97.531 (PAC)	EU281067	*Smilax sp.*	CWD 97.531 (PAC)	EU280992
Spinacia oleracea	N/A	AY453110	*Spinacia oleracea*	no voucher	EU281068	*Spinacia oleracea*	no voucher	EU280993

Table 1. List of species sampled, voucher numbers with herbarium acronyms, and GenBank accession numbers. Sequences obtained from GenBank and DNA obtained from other published studies do not list voucher information.

Staphylea trifoliata	N/A	AY453105	*Staphylea trifoliata*	TJB 354 (WMU)	EU281075	*Staphylea trifoliata*	TJB 354 (WMU)	EU281001
Symphoricarpos orbiculatus	CWD 97.801 (PAC)	EU281122	*Symphoricarpos orbiculatus*	CWD 97.801 (PAC)	EU281069	*Symphoricarpos orbiculatus*	CWD 97.801 (PAC)	EU280994
Tamarix parviflora	N/A	AY453117	*Tamarix parviflora*	DHG 1427 (TEX)	EU281070	*Tamarix parviflora*	DHG 1427 (TEX)	EU280995
Tetrastigma diepenhorstii	N/A	AY453109	*Tetrastigma diepenhorstii*	TJB403 (WMU)	EU281091	*Tetrastigma diepenhorstii*	TJB403 (WMU)	EU281016
Vaccinium uliginosum	N/A	AF421035	*Vaccinium arboreum*	CWD 97.310 (PAC)	EU281025	*Vaccinium arboreum*	CWD 97.310 (PAC)	EU280953
Vitis riparia	N/A	AY453123	*Vitis sp.*	CWD 96.701 (PAC)	EU281071	*Vitis sp.*	CWD 96.701 (PAC)	EU280996
Zamia floridana	N/A	AF197721	*Zamia furfuracea*	N/A	AF020583	*Zamia furfuracea*	LMB 283 (PAC)	EU280998
Zea mays	N/A	UO 9987	*Zea mays*	N/A	AF542203	*Zea mays*	N/A	AY506529
