# Supplementary material for: Mitochondrial DNA suggests at least 11 origins of parasitism in angiosperms and reveals genomic chimerism in parasitic plants
Source: BMC Evol Biol. 2007 Dec 21;7:248. doi: 10.1186/1471-2148-7-248 (PMC2234419; doi:10.1186/1471-2148-7-248)
Supplement: Additional File 2 — Table of voucher numbers and GenBank accession numbers. [file 1471-2148-7-248-S2.pdf]

Table 2. List of species sampled, voucher numbers, and GenBank accession numbers for *coxI* intron. Sequences obtained from GenBank do not have voucher information listed.

Species Name	Voucher Number	*coxI*
Arisaema triphyllum	CWD96.310 (PAC)	AY009426
Asclepias tuberosa	KRAL 70275 (BRIT)	EU281054
Asimina triloba	CWD96.312 (PAC)	AY009433
Brexia madagascarensis	N/A	AJ223413
Cassytha filiformis	CWD97.731 (PAC)	EU281076
Catalpa fargesii	N/A	AJ223411
Clerodendrum trichotomum	N/A	AJ223414
Croton alabamensis	CWD96.303 (PAC)	EU281037
Cucumis sativus	N/A	AJ223416
Cuscuta japonica	no voucher	EU281077
Cynomorium coccineum	Hoder 18/Apr/1996 (PAC)	EU281023
Cytinus ruber	KES2738 (BOL)	EU281022
Dendrophthoe pentandra	SNP15593 (SNP)	EU281073
Digitalis purpurea	N/A	AJ223415
Diospyros virginiana	N/A	AJ223417
Epifagus virginiana	CWD 90.127 (PAC)	EU281078
Frasera caroliniensis	CWD 97.537 (PAC)	EU281038
Hebe subalpina	N/A	AJ223419
Hedychium coronarium	N/A	AJ223426
Heliotropium arborescens	N/A	AJ223425
Hevea brasiliensis	N/A	AJ223436
Hydnora africana	N/A	EU281079
Hydrocotyle rotundifolia	N/A	AJ223424
Ilex opaca	CWD97.656 (PAC)	EU281049
Ilex sp.	N/A	AJ223429
Ipomoea coccinea x quamoclit	JRM97.GRHS1083 (PAC)	EU281050
Jasminum polyanthum	N/A	AJ247607
Lamium sp.	N/A	AJ223428
Lennoa madreporoides	OY6 (PAC)	EU281080
Lepionurus sylvestris	N/A	AJ223439
Malpighia glabra	N/A	AJ223433
Maranta leuconeura	N/A	AJ223432
Mitrastema yamamotoi	CWD 99.11 (SNP)	EU281021
Nerium oleander	N/A	AJ223421
Ombrophytum subterraneum	CWD 94.17 (PAC)	EU281081
Peperomia polybotrya	N/A	X87336
Philodendron oxycardium	N/A	AJ223438
Pholisma arenarium	OY26 (PAC)	EU281083
Pilostyles thurberi (TX)	BLTurner 1999 (TEX)	EU281018
Polygala sanguinea	CWD98.0804 (PAC)	EU281061
Prosopanche americana	OY26 (PAC)	EU281082
Rafflesia pricei	CWD 99.01 (SNP)	EU281020
Rhamnus caroliniana	CWD96.315 (PAC)	EU281063
Rhamnus cathartica	N/A	AJ223422
Rhizanthes lowii	SNP14705 (SNP)	EU281019
Rhus glabra	CWD97.522 (PAC)	EU281065
Symplocos paniculata	N/A	AJ223435
Veronica agrestis	N/A	AJ223427
Vinca rosea	N/A	AJ223423
